# *Smilax china* L. Polysaccharide Alleviates Oxidative Stress and Protects From Acetaminophen-Induced Hepatotoxicity *via* Activating the Nrf2-ARE Pathway

**DOI:** 10.3389/fphar.2022.888560

**Published:** 2022-04-29

**Authors:** Kaiping Wang, Linlin Yang, Jing Zhou, Xianglin Pan, Zihao He, Junxi Liu, Yu Zhang

**Affiliations:** ^1^ Hubei Key Laboratory of Natural Medicinal Chemistry and Resource Evaluation, School of Pharmacy, Tongji Medical College, Huazhong University of Science and Technology, Wuhan, China; ^2^ Department of Pharmacy, Union Hospital, Tongji Medical College, Huazhong University of Science and Technology, Wuhan, China; ^3^ Hubei Province Clinical Research Center for Precision Medicine for Critical Illness, Wuhan, China

**Keywords:** Nrf2-ARE., acetaminophen, oxidative stress, acute liver injury, Smilax china L. polysaccharide

## Abstract

The alleviation of oxidative stress is considered an effective treatment for acetaminophen (APAP)-induced acute liver injury (AILI). However, it remains unknow whether the potential antioxidant *Smilax china* L. polysaccharide (SCLP) protects against AILI. In this study, *in vitro* and *in vivo* experiments were conducted to verify the hepatoprotective effect of SCLP against AILI and explore the potential mechanism. We found that SCLP relieved liver histopathological changes; reversed the levels of alanine aminotransferase (ALT), aspartate aminotransferase (AST), malondialdehyde (MDA) and reactive oxygen species (ROS); reversed the change in liver myeloperoxidase (MPO) activity; and enhanced liver antioxidant (GSH, GSH-Px, and t-SOD) levels in APAP-treated mice, thereby significantly reducing APAP-induced liver toxicity. SCLP rescued the cell viability and alleviated oxidative stress in H_2_O_2_-treated mouse AML12 (Alpha mouse liver 12) hepatocytes. The results of the mechanistic studies showed that SCLP upregulated nuclear factor E2 related factor (Nrf2) expression, promoted Nrf2 nuclear translocation, and enhanced the ability of Nrf2 to bind antioxidant response elements (AREs). Furthermore, SCLP activated Nrf2-ARE pathway, thus upregulating the expression of oxidative stress-related proteins heme oxygenase 1(HO-1), NAD(P)H quinone dehydrogenase 1(NQO-1) and glutamic acid cysteine ligase catalytic subunit (GCLC). In conclusion, this study confirmed the close correlation between liver protection by SCLP upon exposure to APAP and activated of the Nrf2-ARE pathway. These findings suggest that SCLP is an attractive therapeutic candidate drug for the treatment of AILI.

## Introduction

Acetaminophen (APAP) is the most commonly used antipyretic and analgesic drug and is widely used in clinics. Although APAP is safe at the therapeutic dose, damage caused by this drug due to individual differences must be considered. It has been reported that in the United States alone, APAP accounts for 46% of cases of acute liver injury each year, accounting for a greater percentage of acute livery injury cases than all prescription drugs combined (Jaeschke et al., 2020; Bi et al., 2021). Currently, N-acetyl-L-cysteine (NAC) is approved for the treatment of APAP-induced liver injury due to its ability to promote GSH synthesis, scavenge excess N-acetyl-p-amino-benzoquinone imine (NAPQI) and subsequently reduce protein adduct formation. However, the narrow treatment window limits the clinical application of NAC. Evidence shows that when the treatment window has passed, NAC is unable to reverse liver injury and may even seems to have obvious side effects (Saito et al., 2010; Wang et al., 2016; Jaeschke et al., 2020). Therefore, it is necessary to develop a medicine with a longer treatment window and less toxicity than NAC.

Oxidative stress plays an important role in the occurrence and development of APAP-induced acute liver injury (AILI) (Du et al., 2016; Fan et al., 2018). Under normal conditions, the free radicals generated by APAP metabolism are rapidly removed by GSH and other antioxidant enzymes (Jaeschke et al., 2020). However, in the presence of excess APAP, free radicals accumulate in cells, GSH and other antioxidant enzymes are consumed. Furthermore, excess NAPQI binds mitochondrial proteins and causes mitochondrial oxidative stress, eventually leading to acute liver injury (Yan et al., 2018).

Nuclear factor carotenoid 2-related factor 2 (Nrf2) is a transcription factor that can promote the transcription of various cell protective genes in response to oxidative and electrophilic stress (Wang et al., 2016). In response to oxidative stimuli, Nrf2 binds antioxidant response elements (AREs), thereby initiating the transcription of downstream target genes, such as heme oxygenase-1 (HO-1), glutamic acid cysteine ligase catalytic subunit (GCLC) and NADPH quinineoxidoreductase-1 (NQO-1)(Piper et al., 2005; Wu et al., 2019). Given its regulatory effect on oxidative stress in the liver, Nrf2 may be a promising target for attenuating AILI.

*Smilax china* L., which is included in the Pharmacopoeia, is a commonly used clinical medicine with a long history in the treatment of pelvic inflammatory disease (Zhang et al., 2021). Extensive previous studies investigating *Smilax china* L. revealed its immunosuppressive (Jiang and Xu, 2003), hypoglycemic (Nguyen et al., 2020), antioxidant (Zhang et al., 2009), and anti-inflammatory (Zhang et al., 2019) activities. We previously purified the pectin polysaccharide SCLP3-2 (abbreviated SCLP herein) from *Smilax china* L. and determined its structure, laying a foundation for studies investigating the pharmacological activity of SCLP (Zhang et al., 2019). Recent mechanistic studies showed that pectin polysaccharides have good antioxidant activity; Similarly, some studies have shown that the water extract of *Smilax china* L. also has antioxidant activity. These studies prompted us to evaluate whether SCLP can affect the Nrf2-ARE signaling pathway to improve the antioxidant capacity. Upon establishing an *in vivo* model of AILI and an *in vitro* model of H_2_O_2_-induced oxidative stress in AML12 cells, in this study, we demonstrated the protective effects of SCLP on against AILI through the Nrf2-ARE pathway. These results suggest that SCLP is a potential drug for the treatment of AILI.

## Materials and Methods

### Reagents and Chemicals

APAP (purity > 99%) was obtained from Shanghai Aladdin Biochemical Technology Co., Ltd. (Shanghai, China). The chemicals 3-(4,5-dimethylthiazol-2-y1)-2,5-diphenyltetrazolium bromide (MTT) and dimethyl sulfoxide (DMSO) were purchased from Sigma Chemical Co. (St. Louis, MO, United States). DCFH-DA and the kits used to measure alanine aminotransferase (ALT), aspartate aminotransferase (AST), glutathione (GSH), malondialdehyde (MDA), glutathione peroxidase (GSH-Px), myeloperoxidase (MPO), total superoxide dismutase (t-SOD), lactate dehydrogenase (LDH) activity were purchased from Nanjing Jian Cheng Bioengineering Institute (Nanjing, China). TRIzol reagent was obtained from Invitrogen (United States). Antibodies against Nrf2, HO-1, GCLC, NQO-1, β-actin, Lamin B1, Histone H3, and GAPDH were purchased from Abcam (Cambridge, MA, United States). A chemiluminescent electrophoretic mobility shift assay (EMSA) kit and biotin-labeled EMSA probe-ARE were purchased from Beyotime Biotechnology Company (Shanghai, China).

### Preparation and Purity Identification of *Smilax china* L. Polysaccharide

Rhizomes of *Smilax china* L. were purchased from Furen Pharmaceutical Co., Ltd.(Hubei, China), Hubei, China, and authenticated by Professor J.L. Ruan of the Tongji Medical College of Huazhong University of Science and Technology. Crude polysaccharide was extracted from the *Smilax china* L. rhizomes by water extraction and ethanol precipitation and then purified by chromatographic fractionation. Finally, purified SCLP with a molecular weight of 16.8 kDa was obtained (Zhang et al., 2019), and the phenol-sulfuric acid method and *m*-hydroxy diphenyl method were used to measure the sugar content. Nucleic acids and proteins were detected by UV spectrophotometry. The structure of SCLP was detected by Fourier transform infrared (FT-IR) spectroscopy with an infrared spectrophotometer.

### Animals and Ethics Statement

**FIGURE 1:**
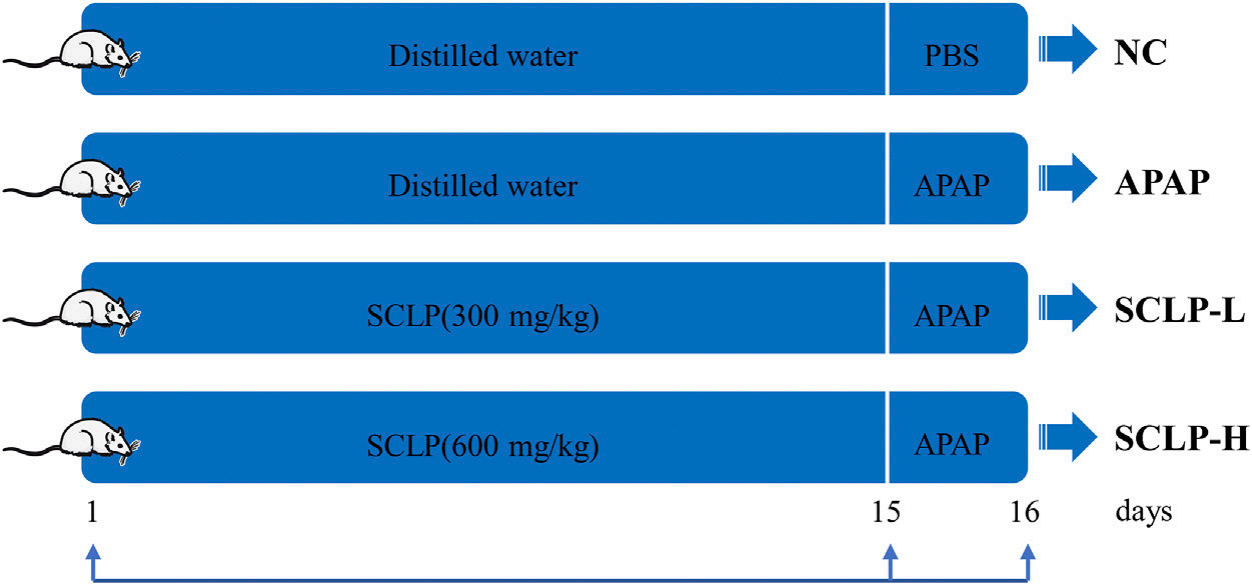
Animal experimental design. NC: normal control group; APAP: APAP model group; SCLP-L: AILI mice treated with SCLP (300 mg/kg); SCLP-H: AILI mice treated with SCLP (600 mg/kg).

Male BALB/c mice (20 ± 2 g) were purchased from the Laboratory Animal Centre of Huazhong University of Science and Technology. The animal experiments were approved by the Animal Research Committee of Tongji Medical College (permit number: SCXK (Hubei) 2016–0057) and conducted in accordance with their guidelines. The experiments were performed according to our pre-experiment study (Supplementary Figure S1) and previously reported study*.* The flow chart of animal experiment is as summarized in Figure 1 (Wei et al., 2018; Shteyer et al., 2019; Pan et al., 2022). After 1 week of adaptive feeding, all experimental animals were randomly divided into four groups (*n* = 8): 1) normal control (NC) group, which was treated with purified water by gavage for 14 days; 2) APAP model group, which was intragastrically perfused with purified water for 14 days; 3) low-dose SCLP (SCLP-L) group, which received SCLP (300 mg/kg) for 14 days via intragastric administration; and 4) high-dose SCLP (SCLP-H) group, which received SCLP (600 mg/kg) for 14 days *via* intragastric administration. On the 15th day, the mice in the NC group received phosphate-buffered saline (PBS) by gavage as a vehicle, and the mice in the other three groups received APAP (400 mg/kg) by gavage. Then mice were sacrificed and liver tissues were removed immediately for subsequent assay, at 24 h after the treatment of APAP. SCLP was dissolved in purified water. During the experiment, all mice were allowed free access to a normal diet and housed at an ambient temperature under a 12 h/12 h light/dark cycle.

### Histological Assessment

After the mice were sacrificed by cervical dislocation, a portion of the liver was removed, fixed in 10% formalin solution, embedded in paraffin, and cut into 5 μm sections. After hematoxylin and eosin (H&E) staining, the sections were observed under a light microscope for histopathological analysis.

### Biochemical Analysis

Serum ALT, AST, LDH, and GSH enzyme activity was measured using assay kits according to the manufacturer’s instructions. GSH-Px, MPO, MDA, and t-SOD levels in mouse liver tissues were determined by commercial kits according to the manufacturer’s instructions.

### Cell Culture and Viability Assay

The nontumorigenic mouse AML12 hepatocyte cell line, was obtained from ATCC (Shanghai, China). The cells were cultured in Ham’s F12 nutrient medium supplemented with 10% fetal bovine serum (FBS) and 1% penicillin-streptomycin, at 37°C in a 5% CO_2_ atmosphere. AML12 cells were preincubated with SCLP for 24 h, and then incubated with H_2_O_2_ (400 μM) or PBS for an additional 2 h. After treatments, the cells were incubated with MTT for 4 h. The generated blue formazan was dissolved in DMSO, and the absorbance was measured at 570 nm.

### Measurement of Hepatic and Cellular Reactive Oxygen Species (ROS) Levels

As reported by Li et al.(Li et al., 2018), hepatic and cellular ROS levels were measured by using the fluorescent dye, DCFH-DA. The staining was analyzed by flow cytometry, and fluorescence images were obtained by laser-scanning confocal microscopy (Nikon).

### Western Blot Analysis

Protein was extracted from mouse liver tissue or AML12 hepatocytes using RIPA lysis buffer. Nuclear and cytoplasmic extracts were prepared using a commercial kit in accordance with the manufacturer’s instructions. The protein samples were separated by SDS-PAGE and then transferred to nitrocellulose membranes. The blots were probed with an appropriate combination of primary antibodies and horseradish peroxidase-conjugated anti-rabbit or anti-mouse IgG secondary antibodies. Finally, the proteins were visualized with an ECL detection system. The band intensities were analyzed by ImageJ, and relative protein expression levels were calculated using β-actin or Lamin B1 as a reference.

### EMSA

Nuclear extracts from liver tissues were prepared with nuclear and cytoplasmic extraction reagents. A gel shift analysis was conducted using an EMSA kit according to the manufacturer’s instructions. Briefly, the binding reactions were performed in a total volume of 10 μL containing 25 μg of nuclear extract, 2 μL of biotin-labeled EMSA probe-ARE, and 5 × binding buffer for 20 min at room temperature. Then, the reaction mixtures were separated on 6% nondenaturing polyacrylamide gels and transferred onto nylon membranes for chemiluminescence detection. In the comp group ,a probe without nucleoprotein was used to verify that the biotin-labeled probe specifically bound Nrf2.

### Quantitative Real-Time PCR

RNA was isolated from cells using TRIzol reagent. The concentration of RNA was measured with a NanoDrop spectrophotometer (Thermo Fisher Scientific, United States). The target gene expression levels were analyzed using the ΔΔCT method and normalization to β-actin mRNA levels (Livak and Schmittgen, 2001). The primer sequences are shown as follows:

NQO-1-F, 5′-CAG​CCA​ATC​AGC​GTT​CGG​TA-3′;
NQO-1-R, 5′-CTT​CAT​GGC​GTA​GTT​GAA​TGA​TGT​C-3′;
HO-1-F, 5′-TGC​AGG​TGA​TGC​TGA​CAG​AGG-3′;
HO-1-R, 5′-GGG​ATG​AGC​TAG​TGC​TGA​TCT​GG-3′;
GCLC-F, 5′-CAG​TCA​AGG​ACC​GGC​ACA​AG-3′;
GCLC-R, 5′-CAA​GAA​CAT​CGC​CTC​CAT​TCA​G-3′;
β-actin-F, 5′-TAT​TGG​CAA​CGA​GCG​GTT​CC-3′;
β-actin-R, 5′-GGC​ATA​GAG​GTC​TTT​ACG​GAT​GTC-3′.

### Statistical Analysis

All results are expressed as the mean ± SD of more than three replicates of each prepared sample. GraphPad Prism version eight was used for the statistical analysis. The data were analyzed by Student’s t test. *p* values less than 0.05 were considered indicated statistical significance.

## Results

### Characterization of *Smilax china* L. Polysaccharide

**FIGURE 2:**
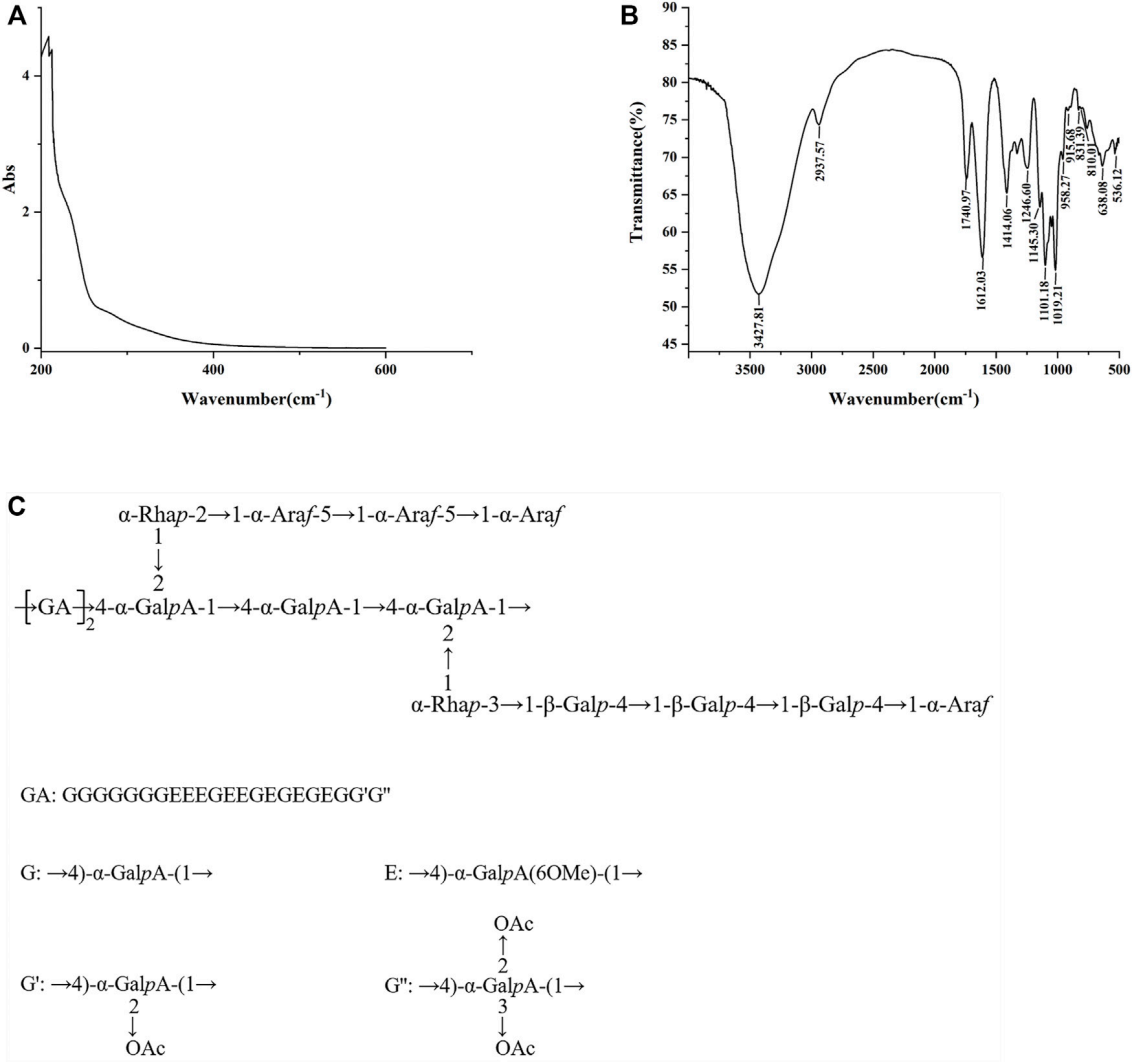
The characterization of SCLP. The UV scanning **(A)** and the FT-IR spectra **(B)** of SCLP. **(C)** The repeating unit of SCLP.

The total sugar content in SCLP was 91.66%, and the content of uronic acid was 85.93%. The UV spectrum of SCLP is presented in Figure 2A. SCLP showed no absorption at 260 nm or 280 nm in the UV spectrum, indicating the absence of nucleic acids and proteins. The FT-IR spectrum of SCLP is shown in Figure 2B. There was no difference between the FT-IR images of SCLP obtained in this study and those we previously obtained, proving that the structure of SCLP is the same as previously reported. The detailed structure of SCLP was elucidated in our preliminary study, and the repeating unit of SCLP is shown in Figure 2C (Zhang et al., 2019).

### *Smilax china* L. Polysaccharide Alleviated Acute Liver Injury in the AILI Mouse Model

**FIGURE 3:**
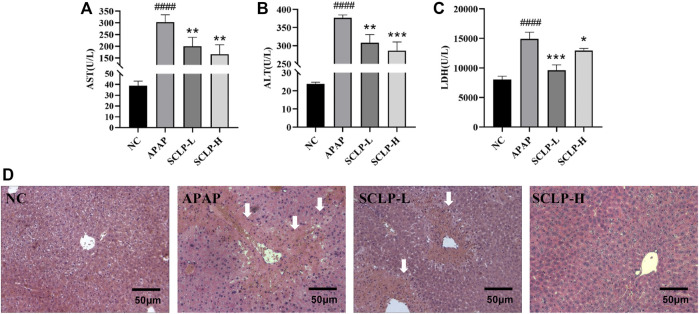
SCLP alleviated APAP-induced hepatotoxicity *in vivo*. Effects of SCLP on serum AST **(A)**, ALT **(B)**, LDH **(C)** in APAP-induced mice. ; **(D)** Liver histological observation (200×). Data are presented as the mean ± S.D. (*n* ≥ 4). ^####^ *p* < 0.0001 vs. NC. *p < 0.05, **p < 0.01, ***p < 0.001, vs. APAP. bar:50 μm.

As shown in Figures 3A–C, the obvious increases in serum AST, ALT and LDH levels induced by APAP were significantly reversed after the administration of SCLP (300 or 600 mg/kg), indicating that SCLP has a hepatoprotective effect. Histological evaluation of the liver further showed that SCLP protected against AILI in the mice, in the APAP group, hepatocytes were irregularly arranged and necrotic, and the liver structure was destroyed. Treatment with SCLP (300 or 600 mg/kg) alleviated these effects, and the necrotic area in liver tissues from the SCLP-treated groups was smaller (Figure 3D). The liver H&E staining results showed that SCLP significantly improved the pathological changes in AILI mouse liver tissue.

### *Smilax china* L. Polysaccharide Alleviated Liver Oxidative Stress in the AILI Mouse Model

**FIGURE 4:**
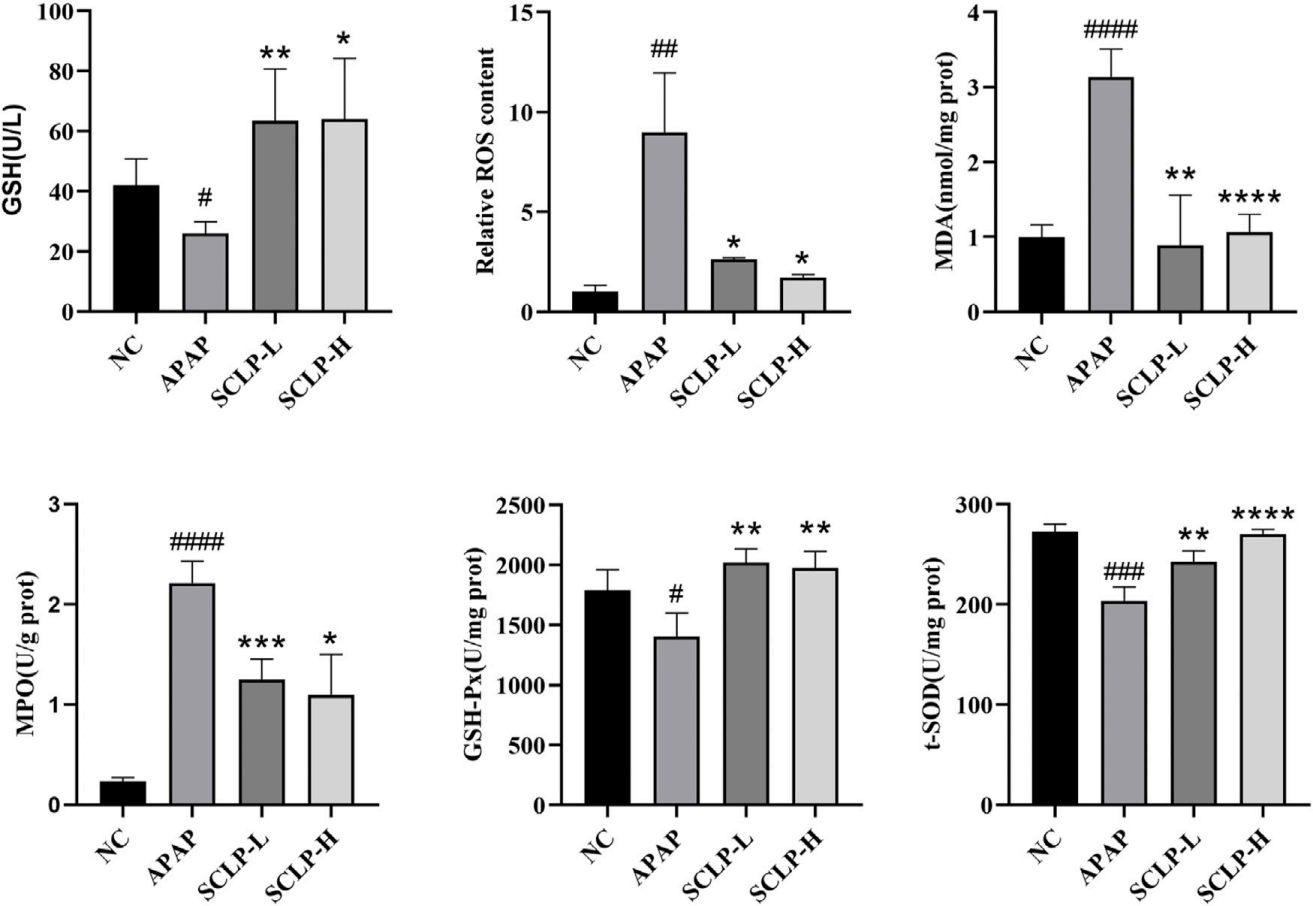
SCLP alleviated liver oxidative stress in AILI model mice. Effects of SCLP on serum GSH **(A)**, liver ROS **(B)**, liver MDA **(C)**, liver MPO **(D)**, liver GSH-Px **(E)**, liver t-SOD **(F)** in APAP-induced mice. Data are presented as the mean ± S.D. (*n* = 4). ^#^ *p* < 0.05, ^##^ *p* < 0.01, ^###^ *p* < 0.001,^#*###*^ *p* < 0.0001 vs. NC. *p < 0.05, **p < 0.01, ***p < 0.001, ****p < 0.0001 vs. APAP.

Oxidative stress has been considered a therapeutic target in AILI (Du et al., 2016). Figures 4A,B show that APAP decreased the GSH content and increased the ROS levels in the mice and that these changes were reversed by the administration of SCLP (300 or 600 mg/kg). In addition, as shown in Figure 4C, MDA levels was significantly decreased after the administration of SCLP, indicating an improvement in liver lipid peroxidation. Figure 4D shows that SCLP effectively inhibited the increase in MPO levels in the liver, thereby alleviating oxidative stress in the AILI mouse model. Furthermore, the contents of GSH-Px and t-SOD were significantly higher in the SCLP treatment groups than in the APAP group (Figures 4E,F), indicating that SCLP can increase the expression of antioxidant factors in the mouse liver.

### Effect of *Smilax china* L. Polysaccharide on Nrf2 Expression and Nuclear Translocation in the AILI Mouse Model

**FIGURE 5:**
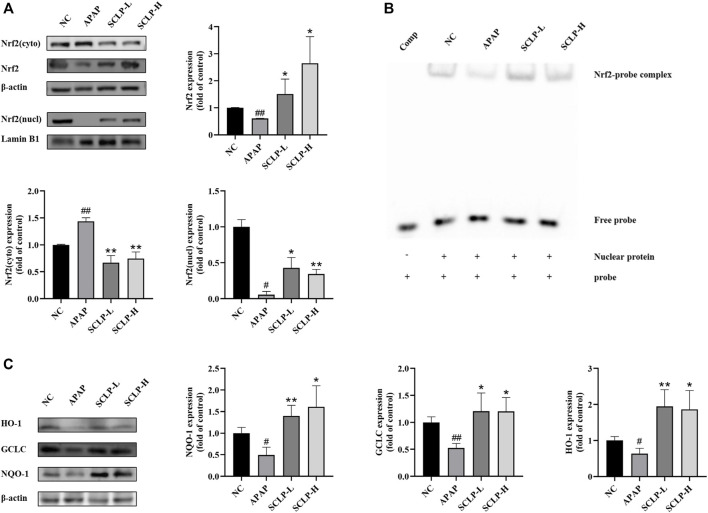
Effect of SCLP on Nrf2-ARE signaling pathway in AILI model mice. Effects of SCLP on Nrf2 protein expression and nuclear translocation **(A)**; Effect of SCLP on the binding activity of the complex of Nrf2/ARE **(B)**; Effect of SCLP on the expression of antioxidant proteins of Nrf2-ARE signaling pathway in AILI model mice **(C)**. Data are presented as the mean ± S.D. (*n* = 3). ^#^ *p* < 0.05, ^##^ *p* < 0.01 vs. NC. *p < 0.05, **p < 0.01 vs. APAP.

The Nrf2-ARE signaling pathway can regulate genes involved in APAP biotransformation and metabolism. Thus, the activation of this pathway is considered a vital therapeutic strategy for the treatment of AILI. As shown in Figure 5A, the expression and translocation of Nrf2 protein were significantly downregulated by APAP compared to the NC. Importantly, these changes were completely reversed by SCLP pretreatment.

### Effect of *Smilax china* L. Polysaccharide on the Binding of Nrf2 to Antioxidant Response Elements in the AILI Mouse Model

To further determine whether SCLP enhances the binding of the nuclear protein Nrf2 to AREs in the mouse liver, nuclear extracts of mouse liver tissue were isolated and subjected to EMSA. As shown in Figure 5B, the gel shift assay identified protein-probe complex formation in all groups except the comp group, suggesting that AREs probe specifically bound the Nrf2 protein. Compared with APAP, SCLP drastically increased Nrf2-ARE binding activity. The EMSA results were consistent with the Western blot analysis results.

### Effect of *Smilax china* L. Polysaccharide on the Expression of Antioxidant Proteins in the Nrf2-Antioxidant Response elements Signaling Pathway in an AILI Mouse Model

The gene expression of the important cellular antioxidative enzymes NQO-1, HO-1 and GCLC was reported to be regulated by the Nrf2-ARE signaling pathway. Therefore, we measured NQO-1, HO-1 and GCLC protein expression levels in the liver. Western blot analysis revealed that the APAP-induced downregulation of NQO-1, HO-1 ,and GCLC expression was markedly reversed by SCLP treatment (Figure 5C).

### Effect of *Smilax china* L. Polysaccharide on Alpha Mouse Liver 12 Cell Viability and Oxidative Stress After Exposure to H_2_O_2_

**FIGURE 6:**
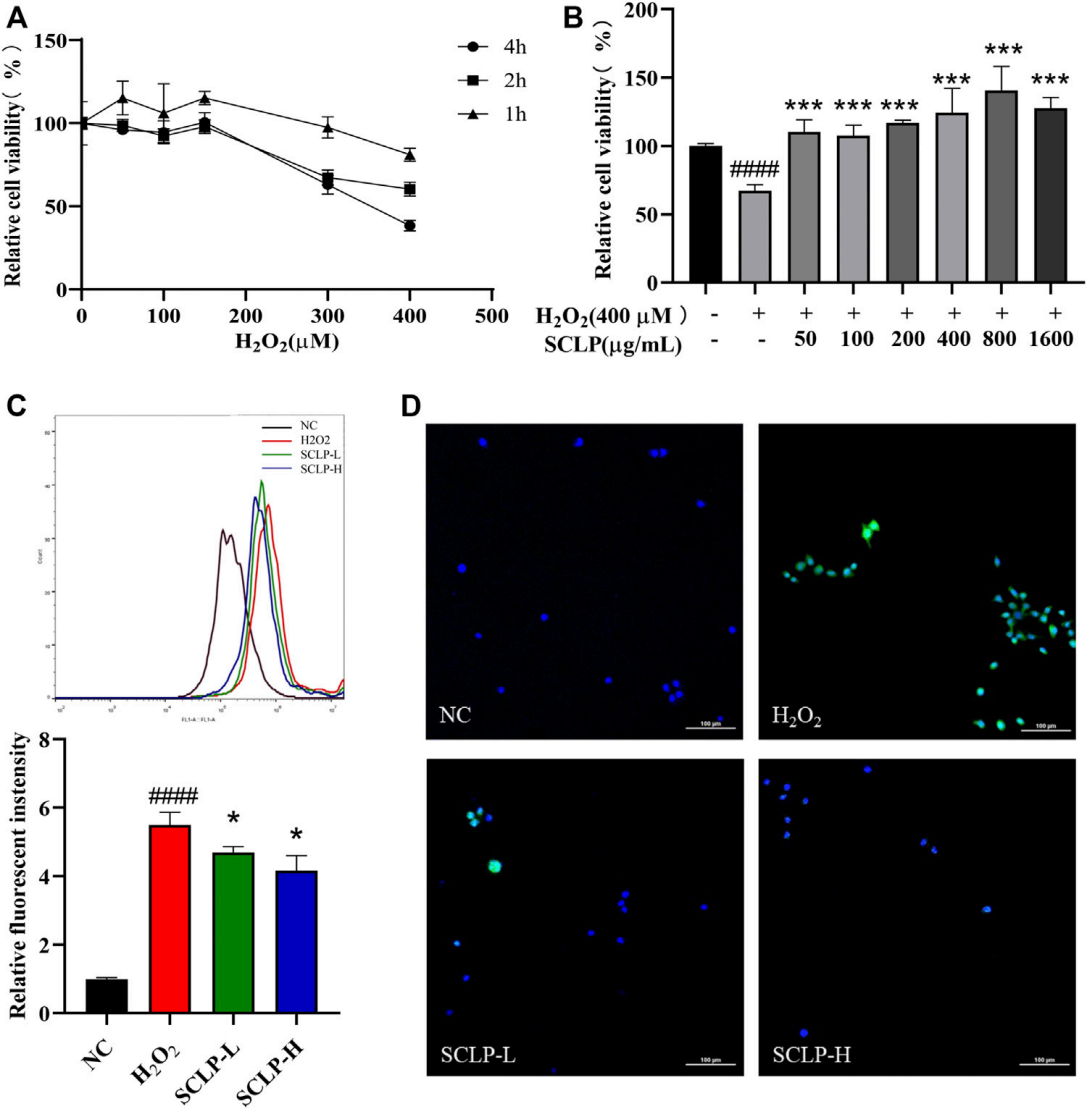
Effect of SCLP on H_2_O_2_-induced oxidative stress in AML12 cells. the effect of different concentrations and incubation time of H_2_O_2_ on AML12 cell viability **(A)**; the effect of SCLP on the activity of H_2_O_2_-induced AML12 cells **(B)**; the effect of SCLP on oxidative stress of AML12 cells induced by H_2_O_2_ (ROS and nuclei are labeled with DCF (green) and DAPI (blue), respectively.) **(C,D)**. Data are presented as the mean ± S.D. (*n* = 6). ^####^ *p* < 0.0001 vs. NC. *p < 0.05, ***p < 0.001 vs. H_2_O_2_. bar: 100 μm.

H_2_O_2_ is regarded as a major mediator trigger of subsequent toxic reactions. H_2_O_2_, the major ROS contributor in cells, is commonly used to evaluate antioxidant capacity (Guo et al., 2020; Gao et al., 2021). Thus, H_2_O_2_-treated AML12 cells were chosen as an *in vitro* oxidative stress model to further elucidate the mechanism by which SCLP alleviates oxidative stress (Cao et al., 2018). Supplementary Figure S4 shown that SCLP have no toxic effect on AML12 cells in the concentration range of 50–1600 μg/mL. As shown in Figures 6A,B, the median effective concentration, a classic measure of drug efficacy, was 400 μM H_2_O_2_ at a treatment time of 2 h. Therefore, the cells were incubated with 400 μM H_2_O_2_ for 2 h to establish the *in vitro* H_2_O_2_-induced oxidative stress model. The H_2_O_2_-induced cytotoxicity was significantly ameliorated by different concentrations of SCLP, among which 400 μg/ml and 800 μg/ml showed the greatest ability to ameliorate H_2_O_2_-induced cytotoxicity. Thus, concentrations of 400 μg/ml and 800 μg/ml SCLP were selected for subsequent experiments.

To further evaluate the antioxidant effect of SCLP on H_2_O_2_-treated AML12 cells, intracellular ROS levels were measured by flow cytometry and laser-scanning confocal microscopy. H_2_O_2_ exposure caused a sharp increase in ROS levels in AML12 cells, and this increase was inhibited by SCLP pretreatment in a concentration-dependent manner (Figures 6C,D). Collectively, these *in vitro* results indicate the therapeutic potential of SCLP in decreasing ROS levels and protecting damaged liver cells.

### *Smilax china* L. Polysaccharide Activated the Nrf2-Antioxidant Response Elements Signaling Pathway and Increased the Transcription and Expression of Antioxidant Enzymes *in vitro*

**FIGURE 7:**
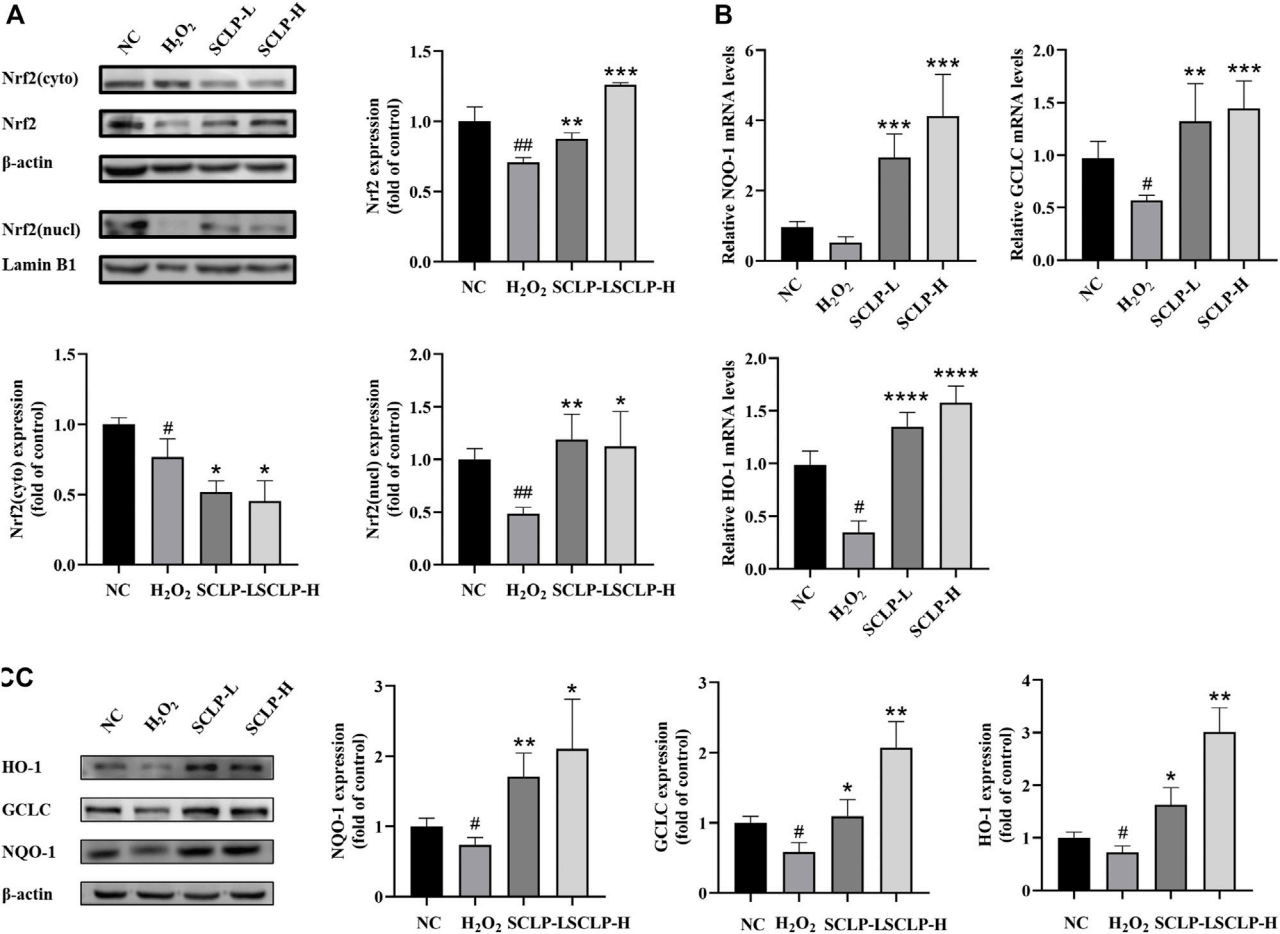
SCLP activated Nrf2-ARE signaling pathway and increased the transcription and expression of antioxidant enzymes *in vitro*. **(A)** the effect of SCLP on expression and nuclear translocation of Nrf2 protein; **(B,C)** the effect of SCLP on translation and expression of NQO-1, GCLC and HO-1 proteins. Data are presented as the mean ± S.D. (*n* = 3). ^#^ *p* < 0.05, ^##^ *p* < 0.01, ^###^ *p* < 0.001 vs. NC. *p < 0.05, **p < 0.01, ***p < 0.001, ****p < 0.0001 vs. H_2_O_2_.

As shown in Figure 7, Nrf2 expression decreased in the nucleus and cytoplasm in AML12 cells after treatment with H_2_O_2_. However, compared with H_2_O_2_ treatment alone, the addition of SCLP pretreatment significantly increased Nrf2 expression. HO-1, NQO-1 and GCLC mRNA and protein levels were significantly decreased in the H_2_O_2_ group compared with the NC group. Moreover, pretreatment with SCLP led to a significant increase in HO-1, NQO-1 and GCLC mRNA and protein levels *in vitro*. In summary, both the *in vivo* and *in vitro* experiments showed that SCLP alleviates oxidative stress by activating the Nrf2-ARE signaling pathway.

## Discussion

The crucial role of mitochondrial oxidative stress in the occurrence and development of APAP-induced hepatotoxicity has been clarified and emphasized for decades. Recent relevant studies confirmed that NAPQI, a toxic metabolite of APAP, is metabolized by cytochrome P450 and covalently binds the sulfhydryl groups of proteins to form NAPQI protein adducts (NAPQI-ADs) after cellular GSH depletion. The generation of NAPQI-ADs in mitochondria is a key step that connects the activation of APAP metabolism to oxidative stress and mitochondrial dysfunction (Fan et al., 2018; Lin et al., 2019). Whether the antioxidant capacity of SCLP contributes to its protection against APAP-induced hepatotoxicity is still not known. The present study aimed to investigate the protective effects of SCLP against APAP-induced hepatotoxicity and further explore the molecular mechanisms *in vivo* and *in vitro*.

ALT and AST are important indicators of liver cell damage and necrosis. ALT and AST activity in blood can double in the presence of a small number of necrotic hepatocytes. Thus, transaminase is a sensitive marker of acute hepatocyte damage (Brodsky et al., 2009). Furthermore, the level of LDH, an important metabolic enzyme related to energy balance in the body, not only changes in the liver, but also increases in the serum in the context of liver disease. In this study, the results indicate that treatment with SCLP reversed the APAP-induced increases in serum AST, ALT and LDH activity and ameliorated hepatocyte degeneration and inflammatory cell infiltration.

GSH, GSH-Px, and t-SOD are critical antioxidants involved in APAP hepatotoxicity. Excessive oxidative stress induces lipid peroxidation, resulting in the destruction of cellular components and cell death (Sun et al., 2018). MDA is a lipid peroxidation marker. An increase in MPO levels is associated with oxidative stress, as this enzyme promotes the production of ROS and reactive nitrogen species (RNS). The inhibition of MPO, which alleviates oxidative stress, may be another therapeutic target (Chen et al., 2020). The above results indicate that along with restoring GSH and antioxidant enzyme (GSH-Px, MPO, and t-SOD) activity in the liver, SCLP significantly inhibited the APAP-induced increase in ROS levels. Moreover, the decrease in MDA content also indicates that SCLP inhibited lipid peroxidation. In conclusion, SCLP has a protective effect on liver oxidative stress injury induced by APAP.

Accumulating evidence has demonstrated that the Nrf2-ARE pathway plays an important role in reducing APAP-induced hepatotoxicity. The transcription factor Nrf2, plays an important role in maintaining cellular redox homeostasis and defending against oxidative stress (Abdelmageed et al., 2021). We investigated the critical involvement of Nrf2 in SCLP-mediated protection against AILI. Our results showed that SCLP upregulated Nrf2 protein expression, promoted its nuclear translocation, and markedly enhanced the binding of Nrf2 to AREs. The Nrf2-ARE signaling pathway has been reported to be responsible for regulating the expression of various major phase II antioxidant enzymes, including NQO-1, HO-1, and GCLC, thereby we examined the expression of these three proteins. APAP administration resulted in a decrease in proteins downstream of Nrf2, and these changes were completely reversed by SCLP. These data indicate that SCLP activates the Nrf2-ARE pathway, thereby enhancing the antioxidant defense system to prevent APAP-induced oxidative damage.

Our *in vivo* findings were further confirmed using an *in vitro* model of H_2_O_2_-induced oxidative stress in AML12 cells. The expression of cytochrome P4502E1, the key enzyme required to metabolize APAP into toxic metabolites, is extremely low in AML12 cells (Jadeja et al., 2015). It is commonly used to evaluate antioxidant capacity, particularly for evaluating ROS scavenging capacity in cells (Urrunaga et al., 2015). Therefore, we treated cells with H_2_O_2_, a major mediator of AILI, instead of with APAP to further characterize the mechanism by which SCLP alleviates oxidative stress. Our study results showed that SCLP significantly reduced ROS levels and enhanced cell viability, indicating that SCLP treatment dramatically alleviated H_2_O_2_-induced oxidative injury in hepatocytes. Furthermore, the assessment of SCLP in AML12 cells *in vitro* confirmed that its antioxidant effects are associated with activation of the Nrf2-ARE signaling pathway.

## Conclusion

In summary, this study is the first to report the antioxidative activity and potential mechanism of SCLP against AILI. The present study demonstrated that SCLP exerts hepatoprotective effects against APAP-induced hepatotoxicity in mice. The activation of the Nrf2-ARE signaling pathway, upregulation of antioxidative enzyme expression and alleviation of oxidative damage are suggested to be the major mechanisms by which SCLP prevents the development of APAP-induced liver damage. In future, the therapeutic potential of these observations remains to be further verified by using Nrf2-deficient mice and Nrf2 knockout or knockdown cells. Hence, further investigation is warranted into the role of SCLP in modulating AILI.

## Data Availability

The original contributions presented in the study are included in the article/ **Supplementary Material** , further inquiries can be directed to the corresponding author.

## Supplementary Material

Supplementary Figure S1 Liver histological observation(200×).

Supplementary Figure S2 Effects of SCLP on Liver GSH. Data are presented as the mean ± S.D. (*n*=4). 518 #*p* < 0.05 vs. NC. **p* < 0.05 vs. APAP.

Supplementary Figure S3 The survival curve of experimental animals.

Supplementary Figure S4 Effect of SCLP on AML12 cells **(A)** and HepG2 **(B)** cells viability. Data are 521 presented as the mean ± S.D. (*n*=6). **p* < 0.05, ***p* < 0.01 ****p* < 0.001 vs. SCLP(0 522 μg/mL). ns: no significant.

Supplementary Figure S5 Effect of SCLP on CYP2E1. Data are presented as the mean ± S.D. (*n*=3). #*p* 524 < 0.05 vs. NC.

Supplementary Figure S6 The original gel image of the western blot in animal experiment. **(A)** Nrf2, 526 **(B)** Nrf2 (cyto), **(C)** Nrf2 (nucl), **(D)** NQO-1, **(E)** GCLC, **(F)** HO-1.

Supplementary Figure S7 The original gel image of the western blot in cell experiment. **(A)** Nrf2, **(B)** 528 Nrf2 (cyto), **(C)** Nrf2 (nucl), **(D)** NQO-1, **(E)** GCLC, **(F)** HO-1.

## Abbreviations

APAP, Acetaminophen; AREs, Antioxidant response elements; AST, Aspartate aminotransferase; ALT, Alanine aminotransferase; DCFH-DA, 2,7-Dichorodi-hydrofluorescein diacetate; EMSA, Electrophoretic mobility shift assay; GCLC, Glutamic acid cysteine ligase catalytic subunit; GSH, Glutathione; GSH-Px, Glutathione peroxidase; HO-1, Heme oxygenase 1; LDH, Lactate dehydrogenase; MDA, Malondialdehyde; MPO, Myeloperoxidase; NQO-1, NAD(P)H quinone dehydrogenase 1; Nrf2, Nuclear factor E2 related factor; ROS, Reactive oxygen species; SCLP, Smilax china L. polysaccharide; t-SOD, Total superoxide dismutase.

## References

[B1] Abdelmageed N. Twafik W. A. Seddek A. L. Morad S. A. F. (2021). Vinpocetine-based Therapy Is an Attractive Strategy against Oxidative Stress-Induced Hepatotoxicity *In Vitro* by Targeting Nrf2/HO-1 Pathway. EXCLI J. 20, 550–561. 10.17179/excli2021-3463 34121971 PMC8192879

[B2] Bi Y. Li Q. Tao W. Tang J. You G. Yu L. (2021). Ginsenoside Rg1 and Ginsenoside Rh1 Prevent Liver Injury Induced by Acetaminophen in Mice. J. Food Biochem. 45, e13816. 10.1111/jfbc.13816 34155666

[B3] Brodsky M. Hirsh S. Albeck M. Sredni B. (2009). Resolution of Inflammation-Related Apoptotic Processes by the Synthetic Tellurium Compound, AS101 Following Liver Injury. J. Hepatol. 51 (3), 491–503. 10.1016/j.jhep.2009.04.024 19595469

[B4] Cao P. Sun J. Sullivan M. A. Huang X. Wang H. Zhang Y. (2018). Angelica Sinensis Polysaccharide Protects against Acetaminophen-Induced Acute Liver Injury and Cell Death by Suppressing Oxidative Stress and Hepatic Apoptosis *In Vivo* and *In Vitro* . Int. J. Biol. Macromol 111, 1133–1139. 10.1016/j.ijbiomac.2018.01.139 29415408

[B5] Chen S. Chen H. Du Q. Shen J. (2020). Targeting Myeloperoxidase (MPO) Mediated Oxidative Stress and Inflammation for Reducing Brain Ischemia Injury: Potential Application of Natural Compounds. Front. Physiol. 11, 433. 10.3389/fphys.2020.00433 32508671 PMC7248223

[B6] Du K. Ramachandran A. Jaeschke H. (2016). Oxidative Stress during Acetaminophen Hepatotoxicity: Sources, Pathophysiological Role and Therapeutic Potential. Redox Biol. 10, 148–156. 10.1016/j.redox.2016.10.001 27744120 PMC5065645

[B7] Fan X. Lv H. Wang L. Deng X. Ci X. (2018). Isoorientin Ameliorates APAP-Induced Hepatotoxicity via Activation Nrf2 Antioxidative Pathway: The Involvement of AMPK/Akt/GSK3β. Front. Pharmacol. 9, 1334. 10.3389/fphar.2018.01334 30546306 PMC6279939

[B8] Gao Y. Shi W. Yao H. Ai Y. Li R. Wang Z. (2021). An Integrative Pharmacology Based Analysis of Refined Liuweiwuling against Liver Injury: A Novel Component Combination and Hepaprotective Mechanism. Front. Pharmacol. 12, 747010. 10.3389/fphar.2021.747010 34630116 PMC8493075

[B9] Guo F. Zhuang X. Han M. Lin W. (2020). Polysaccharides from Enteromorpha Prolifera Protect against Carbon Tetrachloride-Induced Acute Liver Injury in Mice via Activation of Nrf2/HO-1 Signaling, and Suppression of Oxidative Stress, Inflammation and Apoptosis. Food Funct. 11 (5), 4485–4498. 10.1039/d0fo00575d 32378684

[B10] Jadeja R. N. Urrunaga N. H. Dash S. Khurana S. Saxena N. K. (2015). Withaferin-A Reduces Acetaminophen-Induced Liver Injury in Mice. Biochem. Pharmacol. 97 (1), 122–132. 10.1016/j.bcp.2015.07.024 26212553 PMC5909697

[B11] Jaeschke H. Akakpo J. Y. Umbaugh D. S. Ramachandran A. (2020). Novel Therapeutic Approaches against Acetaminophen-Induced Liver Injury and Acute Liver Failure. Toxicol. Sci. 174, 159–167. 10.1093/toxsci/kfaa002 31926003 PMC7098369

[B12] Jiang J. Xu Q. (2003). Immunomodulatory Activity of the Aqueous Extract from Rhizome of Smilax Glabra in the Later Phase of Adjuvant-Induced Arthritis in Rats. J. Ethnopharmacol 85 (1), 53–59. Pii S0378-8741(02)00340-9. 10.1016/s0378-8741(02)00340-9 12576202

[B13] Li H. Chen Y. Zhang J. Chen X. Li Z. Liu B. (2018). Shikonin Attenuates Acetaminophen-Induced Hepatotoxicity by Upregulation of Nrf2 through Akt/GSK3β Signaling. Molecules 24 (1). 10.3390/molecules24010110 PMC6337349 30597971

[B14] Lin L. Guan H. Li R. Liao X. Zhao F. Wang M. (2019). Auriculatone Sulfate Effectively Protects Mice against Acetaminophen-Induced Liver Injury. Molecules 24 (20). 10.3390/molecules24203642 PMC6832223 31600996

[B15] Livak K. J. Schmittgen T. D. (2001). Analysis of Relative Gene Expression Data Using Real-Time Quantitative PCR and the 2(-Delta Delta C(T)) Method. Methods 25 (4), 402–408. 10.1006/meth.2001.1262 11846609

[B16] Nguyen P. T. M. Ngo Q. V. Nguyen M. T. H. Quach L. T. Pyne S. G. (2020). Hypoglycemic Activity of the Ethyl Acetate Extract from Smilax Glabra Roxb in Mice: Biochemical and Histopathological Studies. Iran J. Basic Med. Sci. 23 (12), 1558–1564. 10.22038/ijbms.2020.46658.10763 33489029 PMC7811822

[B17] Pan X. Wang H. Zheng Z. Huang X. Yang L. Liu J. (2022). Pectic Polysaccharide from Smilax china L. Ameliorated Ulcerative Colitis by Inhibiting the galectin-3/NLRP3 Inflammasome Pathway. Carbohydr. Polym. 277, 118864. 10.1016/j.carbpol.2021.118864 34893269

[B18] Piper D. R. Hinz W. A. Tallurri C. K. Sanguinetti M. C. Tristani-Firouzi M. (2005). Regional Specificity of Human Ether-A'-Go-Go-Related Gene Channel Activation and Inactivation Gating. J. Biol. Chem. 280 (8), 7206–7217. 10.1074/jbc.M411042200 15528201

[B19] Saito C. Zwingmann C. Jaeschke H. (2010). Novel Mechanisms of protection against Acetaminophen Hepatotoxicity in Mice by Glutathione and N-Acetylcysteine. Hepatology 51 (1), 246–254. 10.1002/hep.23267 19821517 PMC2977522

[B20] Shteyer E. Ben Ya'acov A. Zolotaryova L. Sinai A. Slae M. Cohen S. (2019). Prevention of Acetaminophen-Induced Liver Injury by Alginate. Toxicol. Appl. Pharmacol. 363, 72–78. 10.1016/j.taap.2018.11.008 30468816

[B21] Sun J. Wen Y. Zhou Y. Jiang Y. Chen Y. Zhang H. (2018). p53 Attenuates Acetaminophen-Induced Hepatotoxicity by Regulating Drug-Metabolizing Enzymes and Transporter Expression. Cell Death Dis 9 (5), 536. 10.1038/s41419-018-0507-z 29748533 PMC5945795

[B22] Urrunaga N. H. Jadeja R. N. Rachakonda V. Ahmad D. McLean L. P. Cheng K. (2015). M1 Muscarinic Receptors Modify Oxidative Stress Response to Acetaminophen-Induced Acute Liver Injury. Free Radic. Biol. Med. 78, 66–81. 10.1016/j.freeradbiomed.2014.09.032 25452146 PMC4392405

[B23] Wang L. Zhang S. Cheng H. Lv H. Cheng G. Ci X. (2016). Nrf2-mediated Liver protection by Esculentoside A against Acetaminophen Toxicity through the AMPK/Akt/GSK3β Pathway. Free Radic. Biol. Med. 101, 401–412. 10.1016/j.freeradbiomed.2016.11.009 27836781

[B24] Wei M. Zheng Z. Shi L. Jin Y. Ji L. (2018). Natural Polyphenol Chlorogenic Acid Protects against Acetaminophen-Induced Hepatotoxicity by Activating ERK/Nrf2 Antioxidative Pathway. Toxicol. Sci. 162 (1), 99–112. 10.1093/toxsci/kfx230 29136249

[B25] Wu C. T. Deng J. S. Huang W. C. Shieh P. C. Chung M. I. Huang G. J. (2019). *Oxidative Medicine And Cellular Longevity* 2019. Artn 9056845. 10.1155/2019/9056845 Salvianolic Acid C against Acetaminophen-Induced Acute Liver Injury by Attenuating Inflammation, Oxidative Stress, and Apoptosis through Inhibition of the Keap1/Nrf2/HO-1 Signaling PMC6535820 31214283

[B26] Yan M. Huo Y. Yin S. Hu H. (2018). Mechanisms of Acetaminophen-Induced Liver Injury and its Implications for Therapeutic Interventions. Redox Biol. 17, 274–283. 10.1016/j.redox.2018.04.019 29753208 PMC6006912

[B27] Zhang Q.-F. Zhang Z.-R. Cheung H.-Y. (2009). Antioxidant Activity of Rhizoma Smilacis Glabrae Extracts and its Key Constituent-Astilbin. Food Chem. 115 (1), 297–303. 10.1016/j.foodchem.2008.11.053

[B28] Zhang Y. Pan X. Ran S. Wang K. (2019). Purification, Structural Elucidation and Anti-inflammatory Activity *In Vitro* of Polysaccharides from Smilax china L. Int. J. Biol. Macromol 139, 233–243. 10.1016/j.ijbiomac.2019.07.209 31376447

[B29] Zhang Y. Zhao Z. Chen H. Fu Y. Wang W. Li Q. (2021). The Underlying Molecular Mechanisms Involved in Traditional Chinese Medicine Smilax china L. For the Treatment of Pelvic Inflammatory Disease. Evid. Based Complement. Alternat Med. 2021, 5552532. 10.1155/2021/5552532 33927774 PMC8052137

