# Supplementary figures and images for: Smilax china L. Polysaccharide Alleviates Oxidative Stress and Protects From Acetaminophen-Induced Hepatotoxicity via Activating the Nrf2-ARE Pathway
Source: Front Pharmacol. 2022 Apr 29;13:888560. doi: 10.3389/fphar.2022.888560 (PMC9098950; doi:10.3389/fphar.2022.888560)

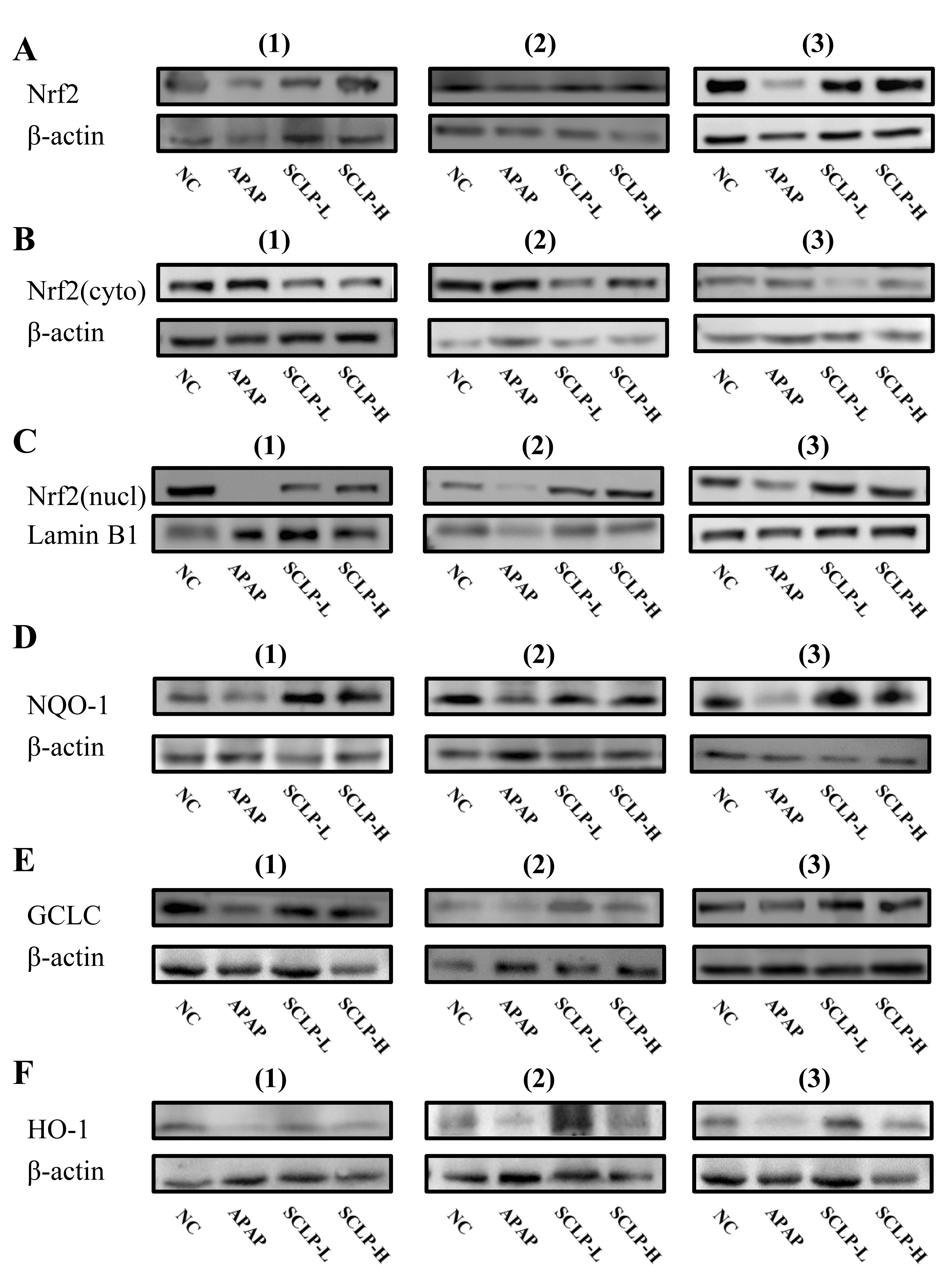

Supplement: Supplementary file 1 [file Image6.TIF]

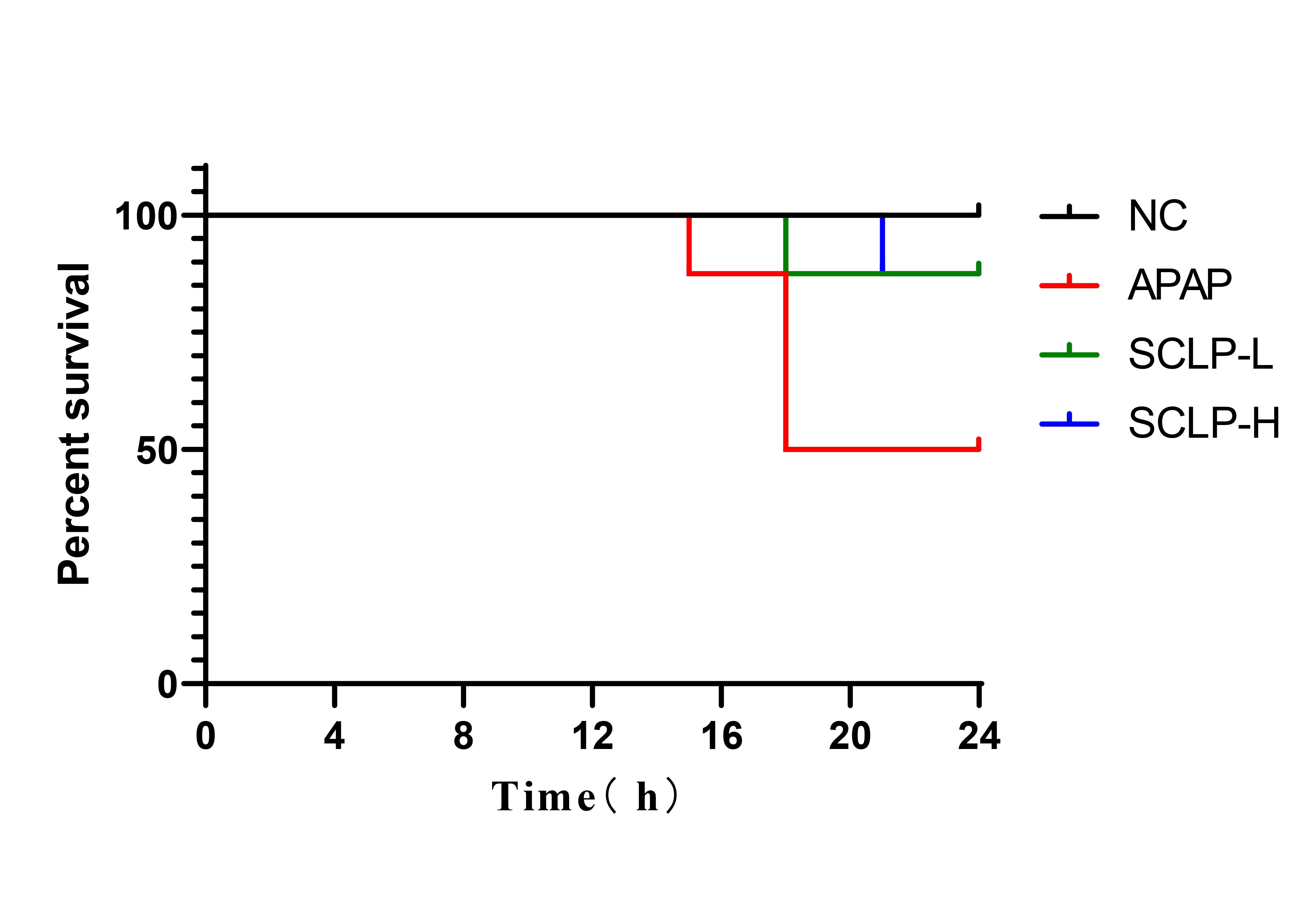

Supplement: Supplementary file 2 [file Image3.TIF]

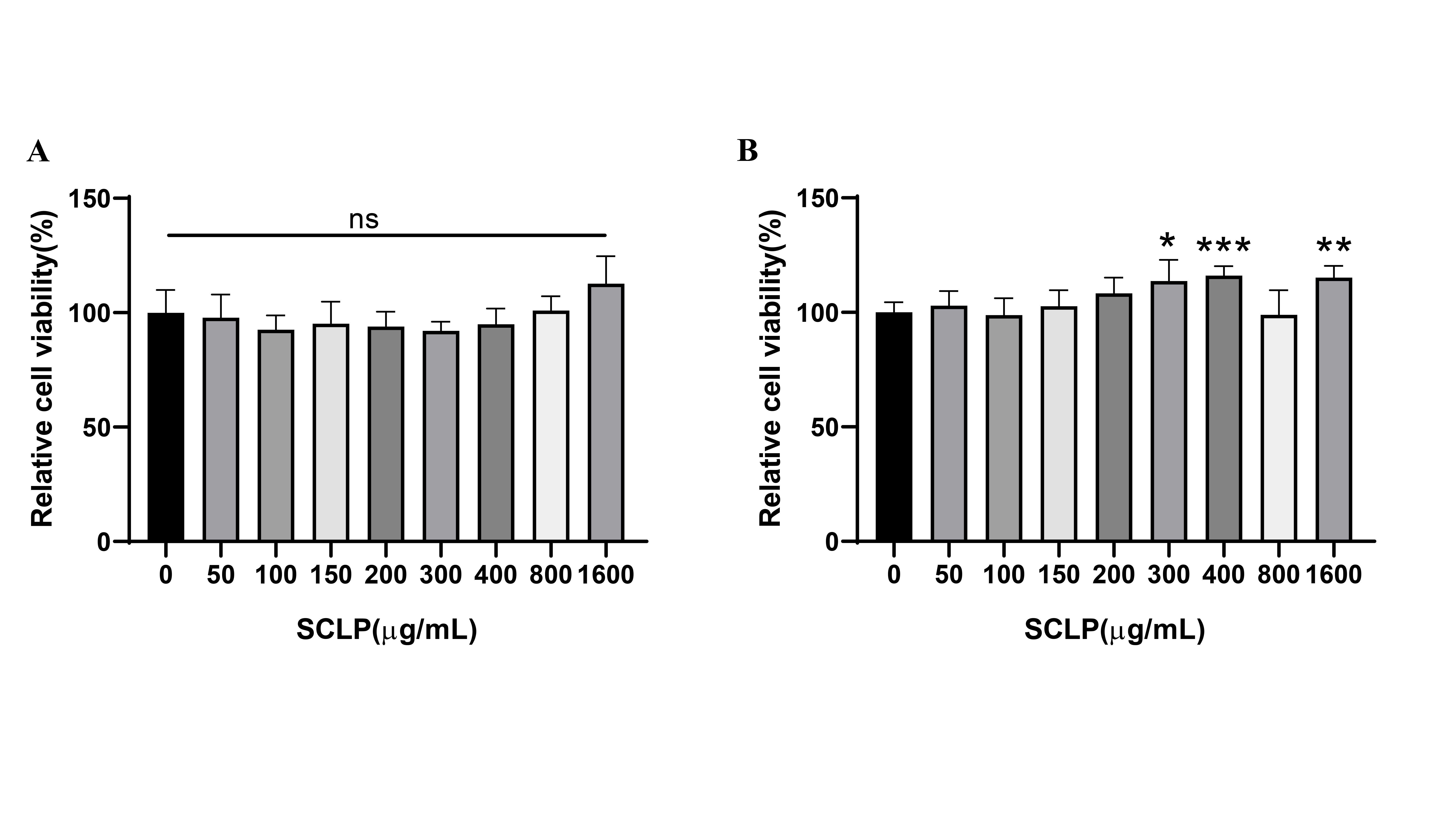

Supplement: Supplementary file 3 [file Image4.TIF]

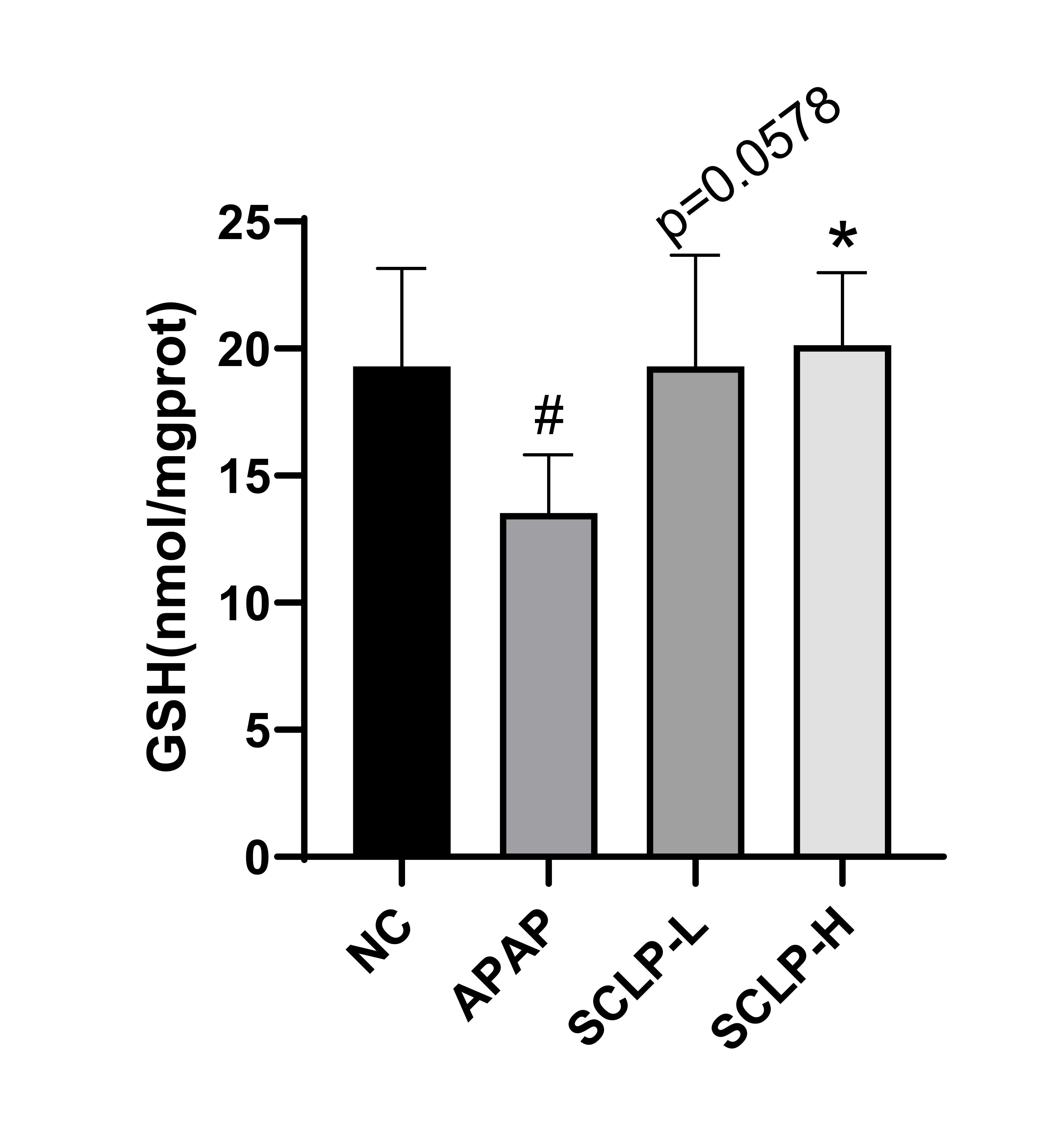

Supplement: Supplementary file 4 [file Image2.TIF]

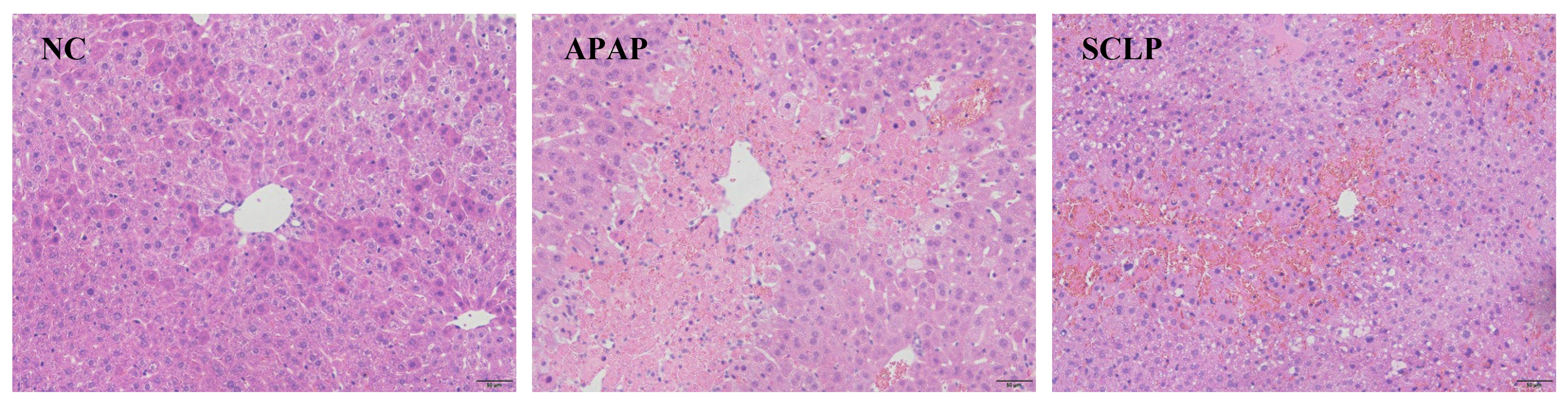

Supplement: Supplementary file 5 [file Image1.TIF]

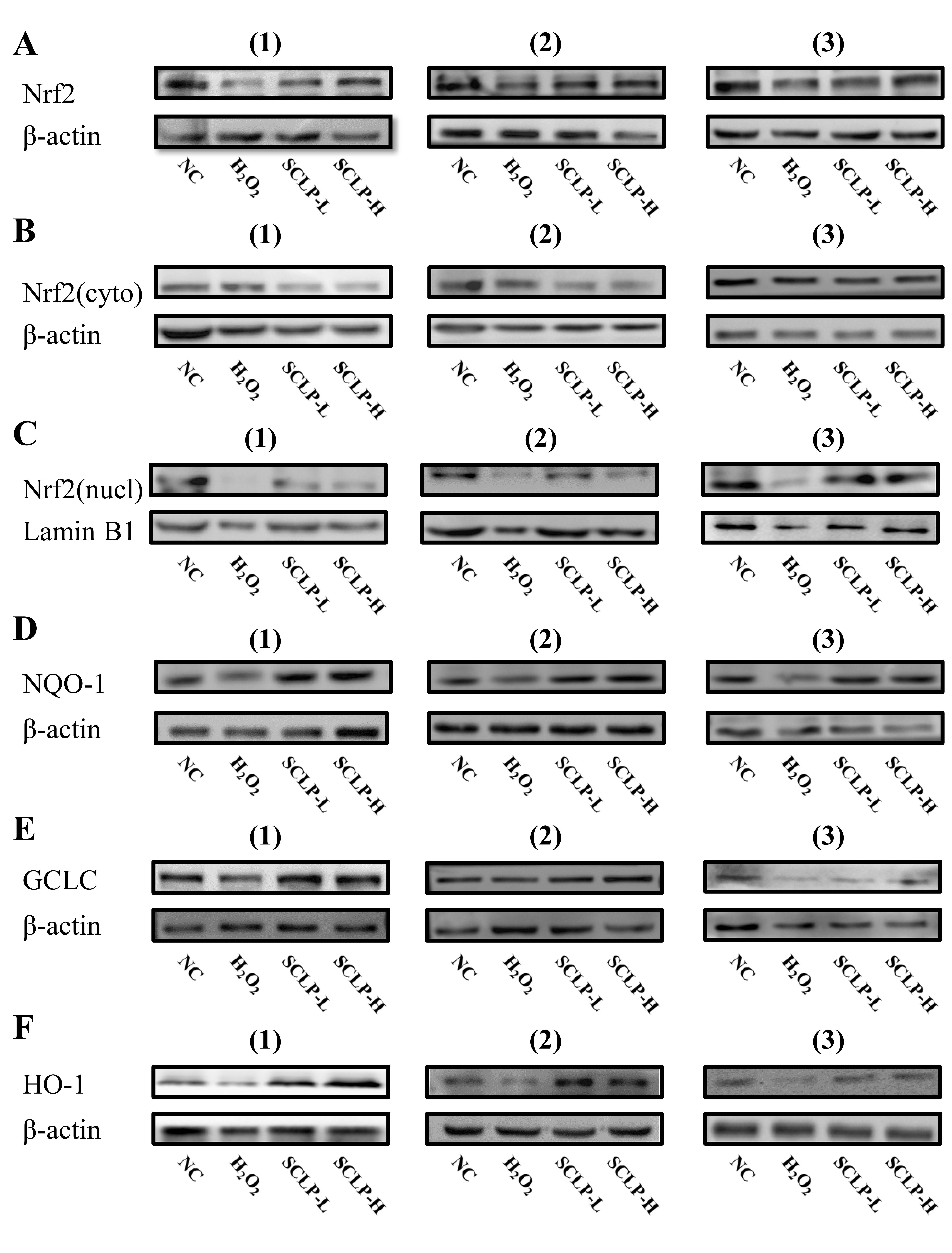

Supplement: Supplementary file 6 [file Image7.TIF]

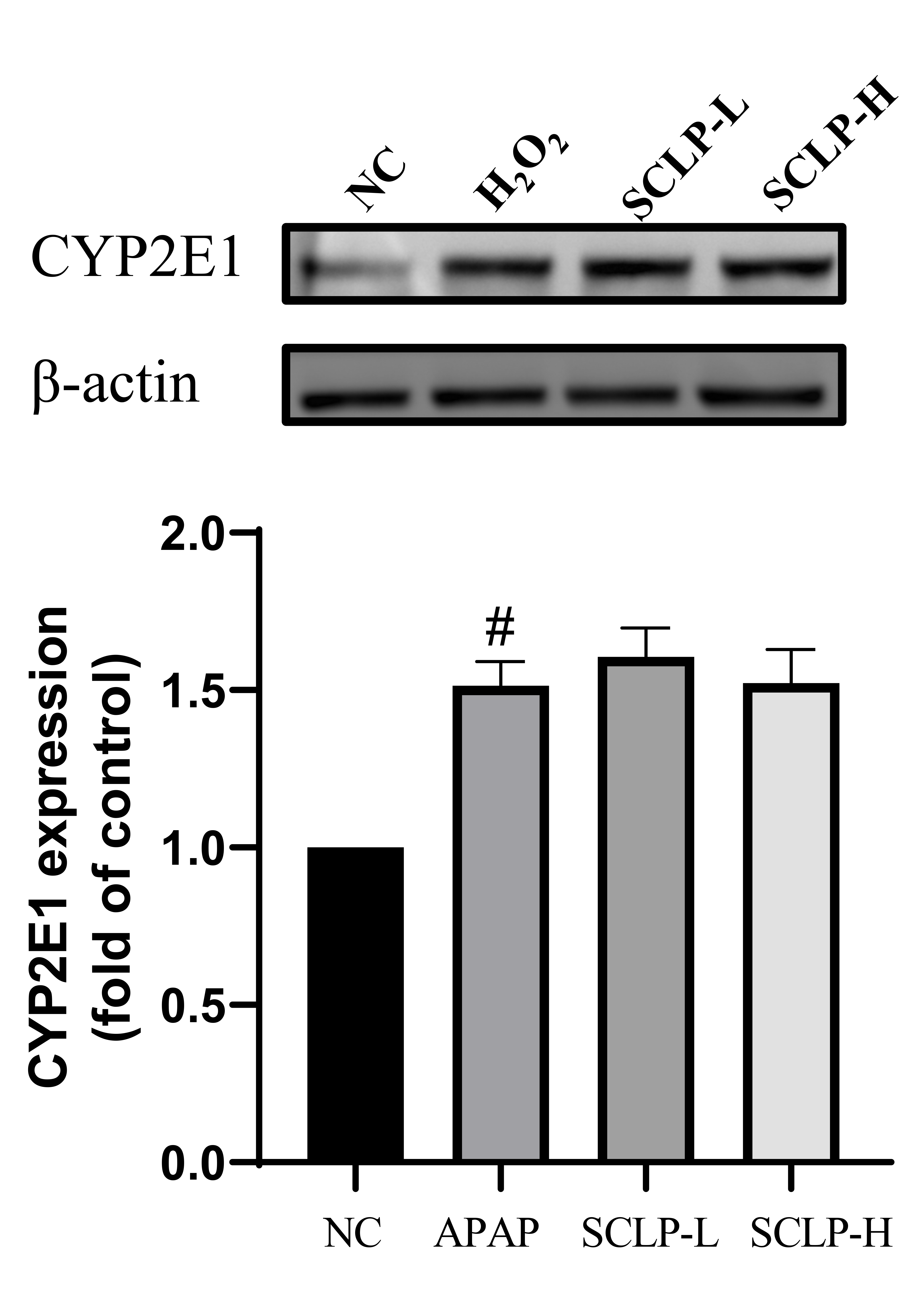

Supplement: Supplementary file 7 [file Image5.TIF]
